# Monthly Summary

**Published:** 1878-02

**Authors:** 


### MONTHLY SUMMARY.

---

*Plating of Iron and Steel with Nickel and Cobalt by Immersion.*—Mr F. Stolba, in a German periodical which we should be glad to give credit to, if there were not six words and fifty-seven letters (including forty-two consonants) in its name—proposes the following simple process for nickel-plating polished iron and steel articles. To a dilute solution (5 to 10 per cent.) of as pure chloride of zinc as possible, there is added enough sulphate of nickel to color it strongly green. This is heated to ebullition in a porcelain vessel. The objects, being completely cleaned of grease, are then suspended in the liquid so that they touch each other as little as may be; and the boiling is kept up for from half an hour to an hour, water being from time to time added in place of that evaporated. The nickel is precipitated in a brilliant white layer wherever the surface of the object is not greasy or rusty. The operation can be continued for several hours if desired; but the plating will not thus be rendered much thicker.

After removing the objects, they are washed with water holding chalk in suspension, and carefully dried. They may afterward be cleaned with chalk, and they take a fine yellowish-toned polish. The chloride of zinc used should contain no metal precipitable by iron. When it cannot be obtained of sufficient purity, it may be made by dissolving zinc scraps in hydrochloric acid, and allowing the solution, containing an excess of metallic zinc, to rest, in order that the metals precipitable by the zinc may separate. Filter at the end of 24 hours, and the solution is ready for use; each portion of zinc dissolved corresponds to about 2,1 parts of chloride of zinc.

The sulphate of nickel should also be as pure as possible, and the cold solution should not precipitate when a plate of iron is plunged in it, as would happen, for example, if it contained copper. When during the operation the liquor becomes a pale green, owing to the precipitation of nickel, more sulphate must be added until the intense green is regained. When the used liquid is exposed to the action of the air, it deposits hydrated oxide of iron, coming from the dissolved metal. It should be filtered, and more chloride of zinc and sulphate added, when it may be again used.

In the same way, polished iron and steel objects may be covered with a brilliant plating of cobalt, by using a sulphate of cobalt solution. The appearance of this plating differs little from that of polished steel. The distinguishing characteristic is the light rose-colored tint. The author states that the plating wears well.—*Scientific American.*

---

*Death from Chloroform Averted by the Inhalation of Nitrite of Amyl.—The British Medical Journal,* August 18th, says: "We have received from a physician the following interesting report for publication: On the 9th instant, I was asked by a professional friend to administer chloroform to a patient of his, from whom he was about to remove a fatty tumor, situated in the left lumbar region. The patient in question was about forty-nine years of age, married, the mother of several children, of thin, spare habit, but otherwise in good health. She was nervous, and apprehensive of the result, entreating me not to give her too much chloroform. Having previously examined the heart and found all the sounds normal, I gave her about two teaspoonfuls of brandy undiluted; and after waiting a few minutes, and placing her in the recumbent posture, I commenced the administration. The chloroform I used was Duncan and Flockhart's upon the purity of which we can always depend. I poured a measured drachm upon a piece of lint, enveloped in a towel. I held it some little distance from her mouth and nose, and let her inhale slowly. My friend noted her pulse, whilst I carefully watched the respiration. The first dose did not produce any effect, and I then used another drachm, which soon caused a good deal of excitement, incoherent talking, and struggling—the patient striving several times to snatch the inhaler from my hand. This gradually subsided, and she appeared to be passing into the third stage of anæsthesia, when she made an abortive attempt to vomit, raised her head from the pillow, and, to my friend's great alarm, the pulse flickered and stopped altogether; she gave a gasp; foam gathered on her lips; her jaw became rigid; and to all appearance she was dead. I immediately withdrew the chloroform; my friend dashed some cold water on her face and pulled her tongue forward, whilst I commenced artificial respiration, after Marshal Hall's method, but without success. We then poured some nitrite of amyl on lint, and held it to her nostrils. In such emergencies, it is impossible to judge the flight of time correctly; but I should say in ten seconds there was a flushing of the face, the pulse was again felt, and, to our great joy, the all-important function of respiration was again restored, the woman being rescued apparently from the very article of death. After a time, the anæthesia seeming tolerably profound, my friend proceeded to remove the tumor, which he did in a rapid and skilful manner, whilst, as the patient grew restless, I gave an occasional whiff of chloroform. It proved to be an ordinary fatty tumor. Only one small vessel required to be ligatured The wound has since healed rapidly, and the patient has made a good recovery. In looking at the array of symptoms, I cannot help forming the opinion that, had it not been for the nitrite of amyl, this poor patient would assurely have died. I have never seen, either in surgical or obstetrical practice, any one in such imminent peril. I am thankful to say I have never witnessed a case of death from chloroform; but, from the accounts published in the medical journals, both I and my friend inferred that, in the present instance, there was syncope arising from paralysis of the heart, and that this was met by the nitrite of amyl, which, in accordance with its physiological effects, gave a direct fillip to the arrested circulation.—*Medical Record.*

*Alumni Meeting.*—The Annual Meeting of the Alumni Association of the Baltimore College of Dental Surgery, will be held at the College Building, on Tuesday Morning, March 5th, 1878, commencing at 10 o'clock. By order of the President.

WM. B. WISE, D. D. S.
*Cor. Secretary.*

---

*Repeated Excision of the Inferior Dental and Gustatory Nerves for the Cure of Dental Neuralgia.*—In a paper read before the Detroit Medical and Library Association, Dr. McGraw relates the following case: The patient suffered from neuralgia of the lower jaw, left side. In February, 1873, Prof. Gross, of Philadelphia, trephined the horizontal portion of the bone and destroyed the nerve. This gave relief for over a year, when the pain returned in the jaw, and made its appearance, for the first time, in the tongue. In June, 1875, Dr. McGraw trephined the ramus of the jaw, and excised about half an inch of the inferior dental and gustatory nerves. After this operation pain remained absent for fourteen months, when it again returned. Acting on the suggestion of Richet, Dr. McGraw then divided the auriculo-temporal nerve, without any effect on the neuralgia. Shortly afterwards he performed the operation recommended by Prof. Gross, and removed the whole of the alveolar process of the left side of lower jaw, with a similar result. Believing that the nerves had become regenerated, he determined to divide them nearer their origins, and to tear them loose from their connections, as is frequently done with such success in neuralgias of traumatic origin. Accordingly, on September 28, 1876, the nerves wer° laid bare and carefully examined, but without finding any break in their continuity at the seat of the former operation. Powerful traction was then applied in the endeavor to loosen them from their attachments, but in vain, and it was found necessary to cut them, about three-quarters of an inch being removed from each. The wound healed rapidly by granulation, and up to this time there has been no return of the neuralgia.—*Detroit Medical Journal.*

---

*Death from Inhalation of Ether.*—We regret to have to record a case in which the administration of ether terminated fatally, and which occurred in the practice of Dr. G. M. Lowe, of Lincoln. The patient was a lady forty-eight years of age, who had discharged the duties of a governess in the family of Dr. Lowe, and had for some time past been suffering from cancer of the breast. A consultation was held, and the removal of the tumor determined upon. The administration of the ether was confined to Dr. Mitchinson, who had large experience in its employment.

All proper precautions appear to have been taken. Dr. Mitchinson examined the heart, and finding it rather feeble, directed the patient to take a little brandy and water. She was quite cheerful, though somewhat nervous. Half an ounce of ether was poured on the inhaler, which was placed over the mouth in the usual way. The valves were open, and gave free ingress and egress to the air. After a few inhalations the patient's face suddenly became turgid and the hands white. The inhaler was at once removed, the tongue brought forward. cold water dashed over the face, and the chest rubbed with brandy; but the breathing became stertorous, the face more and more congested, the pulse failed, there was an effort at vomiting, and death took place in a few seconds. A post-mortem was made by Mr. T. Sympson, the Senior Surgeon of the Lincoln County Hospital, assisted by Mr. T. Brook. On examining the heart, they found that it was feeble and flabby, and some of the tissue, being afterwards examined, was found to have undergone fatty degeneration. The liver was firm, but the whole of its upper surface was attached by old adhesions to the under surface of the diaphragm—the muscular partition between the chest and abdomen. There was a little effusion of serum on the brain. The air cells of the lungs were dilated. The valves of the heart were in a perfectly healthy condition. They also carefully examined the throat, and found nothing there to throw light on the cause of death. The stomach was perfectly healthy; the deceased had not partaken of any food for some time before the ether was administered, which is a point of great importance. Mr. Sympson attributed the fatal result to failure of the heart's action, and the impairment of the functions of the diaphragm in consequence of its attachment to the liver. The fibres of the heart were so feeble as to be unable to bear any extra strain, and the efforts at resuscitation proved abortive owing to the failure of the functions of the diaphragm. Mr. Brook's evidence was to the same effect. They noted, in addition, that the right side of the heart was gorged with blood, that the walls of the right ventricle were very thin, and that there were some nodules of cancer in the liver and lungs.—*Lancet.*

---

*Nitric Acid for Hoarseness.*—Dr. W. Handsell Griffiths (*Canada Medical Record*) says that a few drops of nitric acid in a glass of sweetened water, a couple of times a day, will be found an excellent remedy for the hoarseness of singers. One of the largest fees ever received by him—so he says—was for this prescription.—*Detroit Med. Journal.*

